# The embryo non-invasive pre-implantation diagnosis era: how far are we?

**DOI:** 10.1590/1984-3143-AR2023-0069

**Published:** 2023-09-04

**Authors:** Maite del Collado, Gabriella Mamede Andrade, Natalia Juliana Nardelli Gonçalves, Samuel Fortini, Felipe Perecin, Mateus Maldonado Carriero

**Affiliations:** 1 Science Creating Lives, São Paulo, SP, Brasil; 2 Nilo Frantz Medicina Reprodutiva, Porto Alegre, RS, Brasil; 3 Dasa Genômica, São Paulo, SP, Brasil; 4 Centro de Ciências Naturais e Humanas, Universidade Federal do ABC, Santo André, SP, Brasil; 5 Departamento de Medicina Veterinária, Faculdade de Zootecnia e Engenharia de Alimentos, Universidade de São Paulo, Pirassununga, SP, Brasil; 6 RDO Diagnósticos Médicos, São Paulo, SP, Brasil

**Keywords:** assisted reproduction, embryo, pre-implantation genetic testing, non-invasive, artificial intelligence

## Abstract

Advancements in assisted reproduction (AR) methodologies have allowed significant improvements in live birth rates of women who otherwise would not be able to conceive. One of the tools that allowed this improvement is the possibility of embryo selection based on genetic status, performed via preimplantation genetic testing (PGT). Even though the widespread use of PGT from TE biopsy helped to decrease the interval from the beginning of the AR intervention to pregnancy, especially in older patients, in AR, there are still many concerns about the application of this invasive methodology in all cycles. Therefore, recently, researchers started to study the use of cell free DNA (cfDNA) released by the blastocyst in its culture medium to perform PGT, in a method called non-invasive PGT (niPGT). The development of a niPGT would bring the diagnostics power of conventional PGT, but with the advantage of being potentially less harmful to the embryo. Its implementation in clinical practice, however, is under heavy discussion since there are many unknowns about the technique, such as the origin of the cfDNA or if this genetic material is a true representative of the actual ploidy status of the embryo. Available data indicates that there is high correspondence between results observed in TE biopsies and the ones observed from cfDNA, but these results are still contradictory and highly debatable. In the present review, the advantages and disadvantages of niPGT are presented and discussed in relation to tradition TE biopsy-based PGT. Furthermore, there are also presented some other possible non-invasive tools that could be applied in the selection of the best embryo, such as quantification of other molecules as quality biomarkers, or the use artificial intelligence (AI) to identify the best embryos based on morphological and/or morphokitetic parameters.

## Introduction

Despite the scientific and technological progress obtained in the field of assisted reproduction (AR) in recent years, including improvements in infertility diagnosis tools, better oocyte recovery, and, most drastically, great improvements in embryo in vitro culture conditions, the implantation rates are still lower than expected. The main causes of implantation failures are still poor embryo quality and ploidy alterations (Maxwell and Grifo, 2018). For the selection of the best embryos, the only tool that remains generally available is to perform preimplantation genetic testing for aneuploidy (PGT-A) on trophectoderm biopsies, even though this technique is not indicated for all patients. The main indications for PGT-A in AR cycles are advanced maternal age (usually determined as women above 37 years), history of previous miscarriages, or alterations in karyotype in one of the parents (Sciorio et al., 2020).

In cycles where patients do not have PGT indication, the embryo selection for transfer is usually based on morphology or on morphokinetic parameters, which have shown promising results (Rubio et al., 2014; Meseguer et al., 2012), when a time-lapse incubator system is available. For those reasons, several scientific groups and companies are looking for non-invasive embryo quality biomarkers.

A few years ago, reproductive genetic companies started to develop the non-invasive PGT-A (niPGT-A). The niPGT-A caused a huge repercussion in RA community, since it has the potential to be the first non-invasive tool to analyze the embryo ploidy without the risks associated with trophectoderm biopsy (Xu et al., 2016) along with the perspective of improving the implantation rates for all patients. However, there are still long ways to go and a lot of research to be done before this niPGT-A could be effectively applied in everyday clinical practice. Furthermore, there are different approaches and techniques that could be applied to embryo selection, which is why many researchers are searching for a reliable biomarker or to develop a tool that will dramatically improve embryo selection and, consequently, the current success rates in AR.

Here, we will describe the evolution of pre-implantation diagnosis in AR, from the preimplantation genetic testing (PGT) history to the most recent research. We will also share our view on the perspectives for the non-invasive pre-implantation diagnosis tools.

## PGT: how it started

Preimplantation genetic testing - PGT (formerly known as preimplantation genetic diagnosis – PGD or preimplantation genetic screening - PGS) is widely used today in in-vitro fertilization (IVF) centers over the world for selecting euploid embryos for transfer and to improve clinical outcomes in terms of embryo implantation, clinical pregnancy, and live birth rates (Greco et al., 2020).

In the 90s, chromosome-specific fluorescent in situ hybridization (FISH) was developed and allowed PGT for chromosomal abnormalities (Griffin et al., 1991). However, it was only with the implementation of Next Generation Sequencing (NGS) that PGT increased in popularity in several laboratories worldwide and significantly improved the accuracy of the results (Kane et al., 2016).

Initially, PGT was proposed to be performed on one or two blastomeres biopsied from embryos on day 3 of development. However, improvements in culture medium, the introduction of laser guided biopsy, as well as cryopreservation technique improvements, allowed scientists to experiment with trophectoderm (TE) biopsy, in which 5 to 10 cells are excised from the embryo at blastocyst stage (Boer et al., 2004).

There are increasing evidences of the efficiency of blastocyst biopsy for PGT procedures. According to recent studies, TE biopsies provide highly accurate results, with an inner cell mass (ICM) disomic concordance rate of 99.5% and 97.3% for euploid and aneuploid results, respectively. Furthermore, this technique allows the detection of embryo mosaicism, which was not possible with single cell blastomere biopsy (Sachdev et al., 2020).

Nevertheless, there are some researchers suggesting that PGT (specifically PGT-A) not only does not improve cumulative live birth rates (CLBR) per IVF cycle started, but actually decreases these rates in comparison to cycles without PGT (Mastenbroek et al., 2021; Kucherov et al., 2023; Yan et al., 2021). The results presented by these studies, however, are far from being definitive proof of the lack of utility of PGT, since there are methodological caveats that put their conclusion under debate, which may include the use of younger patients under the recommended age for PGT-A (under 35 to 37 years old) (Kucherov et al., 2023; Yan et al., 2021), or the use of methods that are no longer recognized as the most appropriate for these analyses, such as single blastomere biopsy on D3 (Mastenbroek et al., 2007).

### Types of preimplantation genetic testing

#### PGT-A: preimplantation genetic testing for aneuploidies

A Preimplantation Genetic Testing for Aneuploidies (PGT-A) is used to detect embryos that have the correct number of chromosomes (euploid) and have greater potential to generate a successful pregnancy. This classification, for humans, occurs when the embryos’ cells contain the typical set of 46 chromosomes. An embryo is regarded as “aneuploid” when all its cells contain a particular chromosomal abnormality, such as monosomies or trisomies in autosomes. An embryo is deemed “mosaic” when two or more cell populations with different chromosomal content are present simultaneously. This phenomenon originates from post-zygotic errors of mitosis, such as nondisjunction or anaphase lagging, where sister chromatids fail to segregate correctly among two daughter cells. Embryos with a PGT-A result suggesting mosaicism low level (30 to under 50%) can result in seemingly healthy pregnancies and births, albeit with lower success rates than euploid embryos (Viotti et al., 2021).

#### PGT-M: preimplantation genetic testing for monogenic disorders

Preimplantation genetic testing for monogenic disorders refers to a procedure where embryos are evaluated for single gene genetic disorders for which the disease-related locus have been unequivocally identified prior to implantation. About 20% of all PGT cases involve couples at risk for one or more single gene disorders (Rycke and Berckmoes, 2020).

#### PGT-SR: preimplantation genetic testing for structural rearrangements

Chromosomal rearrangements (balanced translocations) are well known to lead to chromosomally unbalanced gametes, which later turn into unbalanced zygotes. Couples carrying balanced rearrangements are at an increased risk of producing embryos with the incorrect amount of genetic material, and often experience repeated spontaneous abortions (Priya et al., 2018). PGT-SR is used to detect chromosomally normal/balanced embryos, which improve the chance of establishing a healthy pregnancy

## Non-invasive PGTA

Non-invasive preimplantation genetic testing (niPGT), together with morphology and morphokinetics (Navarro-Sánchez et al., 2022), have been proposed as an alternative to the conventional embryo biopsy, which is an invasive procedure to the embryo and requires specialized equipment and highly trained personnel to select the best embryo for transfer in assisted reproduction (Vera-Rodriguez and Rubio, 2017).

Reproductive scientists have dedicated efforts in developing a non-invasive approach to PGT and, in 2013, Stigliani et al. (2013) first confirmed the existence of cell-free DNA (cfDNA) in blastocyst’s spent culture medium (SCM) (Stigliani et al., 2013). From the non-invasiveness perspective and easy handling, blastocyst culture medium would be an ideal source for chromosome screening and, in 2016, the niPGT emerged to analyze DNA released by human embryos into SCM (Xu et al., 2016). In niPGT, the cfDNA is collected from the SCM and sequenced on a next-generation sequencing (NGS) platform, or analyzed by array comparative genomic hybridization (aCGH) following whole-genome amplification (WGA) in the mainstream pipeline (Kuznyetsov et al., 2018)

However, the origin of this cfDNA still remains unexplained. Genomic and mitochondrial DNA contents were identified at SCM (Magli et al., 2016; Stigliani et al., 2013). Regarding the genomic DNA, both blastocyst cell types, trophectoderm (TE) and inner cell mass (ICM), are potential sources. The main cellular mechanisms driving the DNA release into culture media are apoptosis and necrosis (Kuznyetsov et al., 2020), related to embryo development and fragmentation. However, there is growing evidence to support that the cfDNA in SCM is not derived solely from these two embryo mechanisms. If that was the case, aneuploid cells would be over-represented in these samples, which is not what have been reported in recent studies, that observed high concordance rates between SCM and TE or ICM (Huang et al., 2019)

Recently, it was proposed that part of the cfDNA in culture media could be from extracellular vesicles (EV) and these genetic sources are important non-invasive clues of embryo well-being (Hawke et al., 2021b; Tomic et al., 2022). On the other hand, according to Orvieto et al. (2021), 55.5% of euploid blastocysts expel aneuploid debris, suggesting that the primary source of cell-free DNA in culture media is aneuploid blastomere extrusion and/or their fragments as a result of embryo self-correction, therefore, the blastocyst chromosomal diagnosis could be potentially useless (Orvieto et al., 2021). Again, experimental data showing high concordance rates between cfDNA and TE and ICM biopsies do not fully support this conclusion, demonstrating that the origin of cfDNA is still a mystery that needs to be further investigated (Tomic et al., 2022).

**Figure 1:**
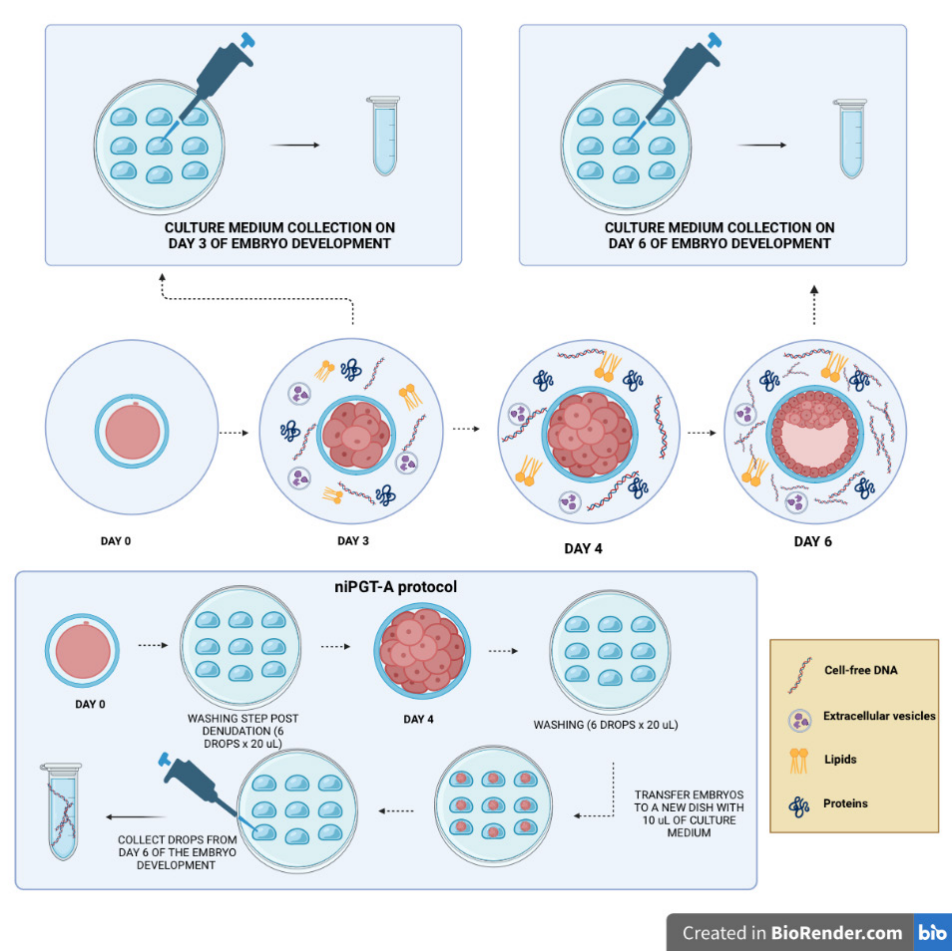
Exploring the non-invasive pre-implantation diagnosis biomarker from the spent media. Graphical scheme representing the workflow for collecting the spent blastocyst medium (SBM) on day 3 and day 6 of in vitro culture to identify biomarkers. Below there is a description of the niPGT-A protocol, with the washing of the embryos to remove cells and contaminants from the culture medium on day 1 and day 4 of culture, and passage of the embryos on day 4 to 10uL microdrops where they remain in development until day 6.

For the success of genetic diagnosis through the niPGT, the laboratory protocols, as well as culture condition and medium retrieval, have to be adapted, and some care is needed to improve the reliability of niPGT (Figure 1). It is important to avoid maternal DNA contamination of culture media by the presence of cumulus cells. So, proper denudation, media changes, and serial washes of embryos before transfer to the culture drop are necessary. Embryos also should be handled individually using new capillaries to avoid cross-contamination (Rubio et al., 2020, 2019). Another concern about the niPGT is the extended embryo culture period until day 6 or 7, since it has already been shown that the use of day 5 embryos provides the best results regarding clinical rates (Bourdon et al., 2019; Yu et al., 2023). This longer culture system is recommended to increase the embryonic cfDNA quantity consequently making a significant difference in concordance rates between TE biopsy, ICM, and whole blastocysts (Rubio et al., 2020, 2019; Chen et al., 2021; Huang et al., 2019). According to Ni-ChinTsai et al., 2022, performing niPGT without altering the daily laboratory procedures cannot provide a precise diagnosis (Tsai et al., 2022).

A multicenter study showed that different ovarian stimulation protocols, culture conditions, and the quality of the embryo do not affect the accuracy of niPGT, and neither does a smaller drop (~ 10 μL) of culture medium (Rubio et al., 2020). The niPGT informativity and concordance rates may be influenced by several factors: the culture day when the medium is collected, contamination with external and/or cumulus cell DNA, and previous manipulation of the embryos (Navarro-Sánchez et al., 2022). Although niPGT has controversial results, high concordance rates between ICM and TE biopsy, and between SCM and whole blastocysts were reported by several groups (Rubio et al., 2020; Xu et al., 2023; Shitara et al., 2021; Xu et al., 2016; Rubio et al., 2019). These concordance rates between SCM and TE biopsy, ICM and whole embryos can be higher than 90% (Navarro-Sánchez et al., 2022), suggesting that, once more solid data are available, niPGT-A has the potential to assist or even replace traditional PGT of TE biopsied cells (Huang et al., 2019; Shitara et al., 2021).

Although already commercially available in clinical practice, and with great potential, there are still many concerns about niPGT that have not yet been addressed and need to be further investigated to consolidate the reliability of the test. Currently, most studies have focused on niPGT for aneuploides, or niPGT-A. However, the next challenge for niPGT, that is already under investigation by some research groups, is to extent the indication not only for genetic assessment of the chromosome copy number but even to do embryonic analysis of segmental rearrangement, for monogenic conditions (Rogers et al., 2021) or diagnostic reassessment of putative mosaicism (Li et al., 2021).

## Non-invasive tools: beyong the PGT

The occurrence of interactive crosstalk between the oviduct and cleavage stage embryos, or between the blastocyst and the endometrium, is well known in the scientific community. Knowing that, and expecting to detect embryo quality and implantation biomarkers, several studies have been performed to isolated a biomarker molecule from day 3 or day 6 of embryo culture (Figure 1).

Some studies aimed to identify and quantify the amino acids released by embryos during in vitro culture (Beardsley et al., 2010). The amount of serine and histidine in spent media at day 3 of culture has been shown to be related to pregnancy potential of the embryo (Huo et al., 2020). Moreover, several proteins were identified in the culture media, such as the Human Leukocyte Antigen G (HLA-G). HLA-G is a trophoblast-released protein that plays a role in inducing immune tolerance during pregnancy (Ferreira et al., 2016). HLA-G was designated as the most promisor biomarker candidate for several years, with many literature evidences describing the relation between its levels in day 3 and day 5 media and blastocyst implantation potential (Radwan et al., 2022; Desai et al., 2006; Katz-Jaffe et al., 2006). Despite several attempts to prove the prediction power of HLA-G quantification in culture media as an embryo quality biomarker, it seems that, in the clinical practice, we do not get the expected results. Recently, another protein is taking HLA-G place as a promisor protein biomarker, the IL-6 (Dominguez et al., 2015; Bori et al., 2021). A preliminary study by Bori et al. (2021) verified that levels of IL-6 in the spent media could predict live birth potential with an AUC of 1.0 (Bori et al., 2021).

Some metabolites have been indicated as potential predictors of good-quality embryos and their capacity to implant. Lactate levels showed significantly higher levels in the media of embryos that did not achieve blastocyst stage, possibly because of the metabolic stress it went through (Motiei et al., 2020). However, in general, the authors agree about glucose consumption and embryo quality, showing that embryos with better morphology classification and embryos that were able to establish pregnancy, consumed more glucose (Ferrick et al., 2020).

In 2019, it was proposed that the higher oxidative status of the embryo spent media, measured by thermochemiluminescence (TCL), was directly correlated with embryo quality and its implantation potential (Alegre et al., 2019). These authors suggested that embryos with better morphology and a higher implantation potential have a more active metabolism and, consequently, increased oxidative status (Tejera et al., 2016; Alegre et al., 2019; Ferrick et al., 2020). However, studies evaluating glucose consumption and oxidative status presented data against the most acceptable metabolic hypothesis, which is the quiet embryo hypothesis (Baumann et al., 2007; Leese et al., 2008; Leese, 2002). Until now, the precise metabolic pattern during embryo development is still unknown, and for that, there is no potential metabolic biomarker available. Lipids are another class of molecules that might have some prediction power in blastulation and pregnancy (Borges et al., 2016), however, the results in this field are still preliminary.

Although the efforts of scientists, until now, the identification of a biomarker molecule able to predict the embryo potential is quite far from clinical practice. The real question is, what is the embryo key message to focus on non-invasive tools? Unfortunately, there is no easy answer for this, since we still do not know if this message is delivered by one molecule, a pathway, or a combination of both.

Besides the secretome, and maybe with the greater success perspectives, the pre-implantation non-invasive researchers are focusing on developing software with artificial intelligence (AI) based on morphology and morphokinetics embryo parameters (Bori et al., 2020). Interestingly, the accuracy of embryo quality prediction is always higher in those studies that used AI alone or in addition to different approaches, than with a possible biomarker molecule.

Initially, the morphology and morphokinetics parameters obtained by Time-Lapse incubator systems were used without AI help and showed that good quality embryos and, more importantly, euploid embryos, have some identifiable patterns, like faster kinetics and fewer blastocyst contractions (Gazzo et al., 2020; Bamford et al., 2022).

Nowadays, several companies are developing very sophisticated software able to predict embryo euploidy with around 80% efficiency (Huang et al., 2021; Liao et al., 2021), and it does not seem far-fetched to think about reaching closer to 100% in the next few years. Although there are differences among animal species (Gutierrez-Adan et al., 2015), these software are confirming that, at least in humans, the belief of “the faster, the better” theory is right, as euploid embryos are, in fact, faster developers than the aneuploid ones, especially regarding the time to initial and expanded blastocyst (Campbell et al., 2013; Bamford et al., 2022). Moreover, the AI of some time-lapse incubators gives a score to each embryo in order to select the one with the better implantation potential. It has been shown by many studies that the score-guided embryo selection is able to improve not only the implantation rate, but also the live birth rate (Ueno et al., 2022; Bori et al., 2022). Until now, in human reproduction, the AI is the only non-invasive pre-implantation tool routinely used in RA cycles.

## How basic science could help

The development of embryo non-invasive biomarkers relies on two major scientific grounds. The first consists of the efficiency of protocols for isolating and detecting biomolecules secreted by the embryos on the spent media. These molecules include proteins, lipids, nucleic acids, or any other compound secreted by the embryo that can be used as an embryo quality-competence biomarker. Thus, relies on the efficiency of analytical tools.

In the last three decades, investigators searching for embryo biomarkers witnessed massive improvements in analytical tools. The limited search for particular biomarkers – morphological characteristics, a single protein, some nucleic acid sequences in a few samples (Fragouli et al., 2012; Bettegowda et al., 2008; Laurinčík et al., 1996; Latham et al., 1992) – evolved to the brute force screening of biomarkers brought by the ‘omics’ era – hundreds of proteins, lipids, or metabolites, thousands of DNA and RNA sequences, coupled with high-throughput analysis and powerful bioinformatics (Liu et al., 2021; Montani et al., 2019; Rubessa et al., 2018).

Despite these breakthroughs in analytical tools, the search for a set of biomarkers, with high sensitivity and specificity, is still incomplete. Figuring out where the ‘perfect’ set of biomarkers is hidden may be achieved with the second fundamental scientific ground, which is the understanding of embryo biology.

The essentials of embryo in vitro culture changed from undefined conditions – media with animal-derived supplements and cell coculture (Thompson et al., 1998; Thibodeaux et al., 1992; Kane and Headon, 1980) – to a more and more defined culture condition – chemically defined media and absence of other cell types (Arias et al., 2022). The search for non-invasive biomarkers came along with the evolution of embryo culture systems from undefined systems to complete chemically-defined systems. Consequently, most of the already identified non-invasive biomarkers were detected in the spent media of embryos cultured under chemically-defined conditions (Hawke et al., 2021a; Navarro-Sánchez et al., 2022; Li et al., 2022; Esmaeilivand et al., 2022).

Some research outputs on embryo biology demonstrate that the embryo is capable of modifying the maternal reproductive tract as early as at the blastocyst stage or even before. The preimplantation embryo presence modulates the transcriptional responses in the oviduct and uterus (Sponchiado et al., 2019; Mazzarella et al., 2021) and the fluid secreted by them in the lumen (Sponchiado et al., 2019). The current understanding is that the embryo communicates with the maternal tract and adjusts the maternal tract environment to favor its own survival since the very initial moments of embryo interaction with the mother.

This new view on embryo biology – i.e. the very early embryo communicating with the maternal tract – opens two new possibilities in the search for non-invasive biomarkers in embryo in vitro production systems. One is represented by screening the messages exchanged between the embryo and the maternal tract. This strategy has already been explored by the isolation of embryo-secreted extracellular vesicles in the spent media and the association of its cargo with embryo development competence (Melo-Baez et al., 2020; Bridi et al., 2021).

The other possibility correlates with the studies aiming to develop a novel generation of embryo in vitro culture systems that incorporate maternal tract components. Such efforts are ongoing and are illustrated by dynamic culture systems including embryo in vitro culture in 3D oviduct-uterus (Gervasi et al., 2020), coculture of embryos and female maternal tract organoids, and embryo culture in microfluidic oviduct-endometrium-on-a-chip. So, instead of searching molecules of the spent media or screening the messages trafficking via extracellular vesicles, the next generation of non-invasive biomarkers might be represented by the responses elicited by the embryos on the adjacent cells incorporated in these novel embryo culture systems.

## Final considerations

**Figure 2:**
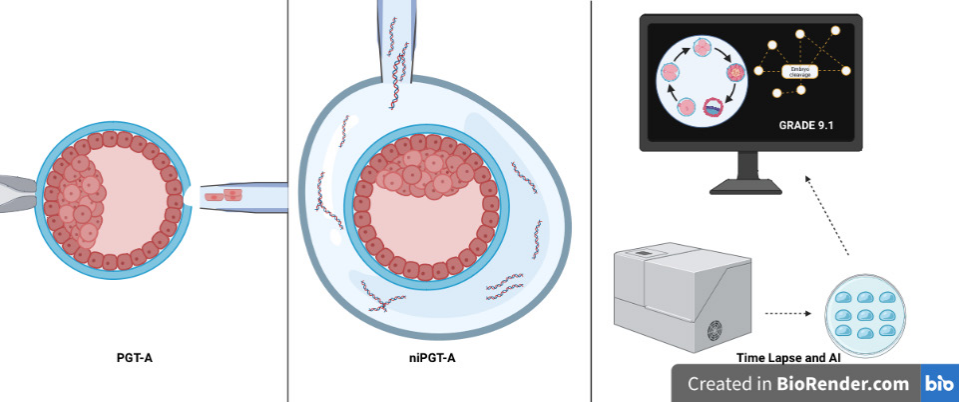
Representation of the pre-implantation diagnosis tools: the invasive PGT-A, the non-invasive niPGT-A and time lapse system with AI.

Bearing in mind that only 30 years have passed since the first pre-implantation diagnosis was developed, the PGT-A, scientists and clinics of RA have reached a substantial success in developing researches for the next step: non-invasive pre-implantation genetic testing to determine embryo quality. The truth is that the results obtained in some of these researches became useful tools to select the best embryo to transfer, such as ni-PGTA or AI (Figure 2). We have to take into account that niPGT-A will not replace PGT-A for those patients that have an indication to undergo aneuploidy testing, but it could be an important tool to help select the embryo to transfer in patients without PGT-A indication that feel the necessity to select the blastocysts with higher implantation potential. However, until know, niPGT-A still costs a significant amount in a process, the RA cycle, that is expensive by itself. Therefore, AI software, in combination with time lapse systems, could be an alternative. The most recent research data with this methodology show that it is able to lead to improvements in the prediction of implantation potential and ploidy status of the embryo, indicating that it will probably be the first non-invasive pre-implantation diagnosis tool capable of reaching larger number of patients in the near future, when compared to niPGT-A. This is in part due to a high number of publications and advantages that we are witnessing about AI, but also because of the lower implementation cost of this methodology in AR laboratories, when compare to niPGT-A.

The better selection of blastocysts will allow us to decreased the implantation failure and miscarriage rates and shorten the patient cycle until they achieve pregnancy. Moreover, the basic science still has the power to answer us some gaps that exist in this field, and approaches, like 3D culture systems or on-a-chip tools, are promising. Once again, is clearer than ever the need of scientific collaboration between basic science researchers and AR clinicians to reach the main goal, which is to make the patient's journey within the AR to get a baby at home as short and gentle as possible.
